# The role of picture books in supporting children’s emotions: perspectives of Saudi kindergarten teachers

**DOI:** 10.3389/fpsyg.2026.1769288

**Published:** 2026-04-30

**Authors:** Norah Saleh Aldossary

**Affiliations:** 1 Department of Kindergarten, College of Education Najran University, Najran, Saudi Arabia; 2 Sharia, Educational and Humanitarian Research Center, Najran University, Najran, Saudi Arabia

**Keywords:** early years education, emotional literacy, kindergarten teachers, picture books, Saudi Arabia, Saudi Vision 2030

## Abstract

**Introduction:**

Emotional connection and behavioral regulation are vital for children’s development. Research shows picture books support emotional and cognitive growth in early childhood. This study examines how picture books contribute to the emotional development of preschool Saudi children, aligning with Saudi Vision 2030 and educational policies, and is guided by four research questions based on Vygotsky’s sociocultural theory.

**Methods:**

A quantitative research design was adopted employing an electronic survey of 113 female kindergarten teachers in Najran city in Saudi Arabia. The survey consisted of 35 validated items. The analysis of the survey was done through descriptive and generalization statistics.

**Results:**

The survey found that teachers in Saudi Arabia use persuasive questioning or engaging narrative for better engagement of students in lessons. The use of picture book narratives and illustrations helped the children recognise and understand some of their emotions through the interactive discussion and mediating strategies adopted by teachers during the reading time. However, the picture books neglected to incorporate topics exploring negative emotions, such as anxiety and anger, which limited opportunities for children to learn about and manage more distressful feelings they might experience.

**Discussion:**

The teachers faced certain obstacles in using picture books for the emotional development of children, including a lack of culturally suitable study materials and guidance in the social and emotional development of children. The study suggests that the use of picture books in lessons be highly promoted for the emotional development of children, while also considering some of the contextual needs relevant to early years education in Saudi Arabia.

## Introduction

Children experience their physical, social, and learning environments in an extraordinary way. To grow and develop in an optimal way, children must learn to identify their feelings and express them freely and without inhibition in requesting support from a carer in a professional or personal capacity when in need of comfort, assistance in understanding their feelings, or support when feeling overwhelmed (Whitburn et al., 2023). If children are denied the chance to express and discuss their feelings, children may experience difficulties in understanding and communicating their feelings as they grow and develop (Papadopoulos, 2021).

The home and school socialization environments are the earliest foundation upon which kindergarten develop their emotional understanding and regulation in most cases. Parents are a significant influence on the emotional development of children through modeling, labeling, and responding to their children’s emotions. Research by Zinsser et al. (2021) revealed that the modeling and responding behavior of parents in the home environment is significant in assisting the development of children through context. Similarly, Silkenbeumer et al. (2024) found in their study that the classroom is a socially controlled environment in which the teacher applies emotion coaching and co-regulation strategies such as affirming the children’s feelings and putting in place scaffolding for regulatory strategies in the development of children’s independent self-regulation skills. All these findings show how children’s emotional development is socially constructed and intentional, as Vygotsky (1978, 1962) posited. Both home and kindergarten are therefore important environments in which children’s emotional learning is deliberate. It is therefore advisable for parents and teachers to create environments in which children are encouraged to engage in reflective dialogues about their emotions and provide guidance in helping children’s development of appropriate strategies in the expression of their emotions in a safe and appropriate way (Papadopoulos, 2021). Therefore, parents and teachers are in a position to play a significant role in helping children learn about their emotions, learn how to express their emotions, and learn how to manage their emotions in a safe and appropriate way. Children who are exposed to positive behavioral models on a regular basis may greatly benefit from such exposure because children are able to learn from what they observe (Liu and Green, 2024).

At the age range of two to four years, the child begins to exhibit empathetic behavior. This empathetic ability may be instilled in the child through the process of reading alone, as well as reading with another child and then discussing the story, where the child is exposed to different characters, emotions, and views (Rangel-Rodríguez et al., 2021; Twiner et al., 2022). Self-awareness is considered one of the basic elements of empathy, which indicates that if the child is aware of their own emotions, they are more likely to empathize with the emotions of other people (Cordeiro et al., 2021; Shoshani, 2024). Furthermore, emotions are more easily expressed by children through non-verbal communication, including body language, which may be an effective means of communication (Kachel et al., 2021; Kleye et al., 2021). It may help parents gain more insight into the actual emotional state of their children through learning how to identify these non-verbal behaviors exhibited by the child (Kleye et al., 2021). Subsequently, applying this knowledge to children’s books and conversations can assist parents and teachers to develop more awareness and guide children away from undesirable behavior as they grow up.

This notion is built on the premise that comprehending emotions is important in satisfying personal needs and determining whether an environment is safe and supportive enough for effective individual functioning (Lanza et al., 2023; LoBue and Ogren, 2022). Therefore, when children lack the skills necessary to identify, understand, and control their emotions, they may struggle with challenges in their lives as they grow older (Malik and Marwaha, 2022; Weir, 2023).

This leads to the concept of an affective environment, which is widely perceived as a blend of emotions derived from various encounters and situations, as well as those experienced through the physical senses, such as movies, cartoons, and novels, presenting certain emotions with a specific amount of intensity (Barrett et al., 2019; Greber et al., 2023; Wang et al., 2023). Thus, children gradually learn how to express emotions by observing and experiencing the feelings they encounter through seeing, hearing, or reading (Marcin, 2020).

Children between the ages of four and 12 begin to develop their range of emotional experiences, informed by their uniquely individual encounters (Cruz et al., 2024; Sanchis-Sanchis et al., 2020; Tan et al., 2022). During this developmental stage, it is instrumental to broaden children’s limited emotional repertoire by introducing them to a wide spectrum of emotions depicted in age-appropriate picture books. These resources serve as effective instructional tools for integrating emotional learning into life skills development (Song, 2024). Sharing stories through pictorial representations that investigate emotions not only prepares children to deal with real-life emotions, but also helps them to create a healthy, positive attitude to their emotional experiences (Song, 2024). These perceptions collectively imply that the emotional foundations formed during childhood have a major influence on the person they will become as they grow.

## Research aim and objectives

Despite growing international interest in children’s emotional development and the use of picture books to support emotional learning, existing research has largely focused on theoretical benefits and pedagogical practices within Western educational contexts (Oberman, 2023). Recent studies in the Kingdom of Saudi Arabia (e.g., Al Najim, 2024; Alsaleh, 2019; Asiri, 2025) have begun to consider the potential and practical methods for use of picture books in early childhood education (ECE) but have yet to delve into the deliberate, curriculum-based application of picture books with emotional themes in kindergarten teacher practices to assist in children’s social and emotional development. Moreover, there has been limited empirical research on the utilisation of picture books by Saudi kindergarten teachers or their practices to promote children’s emotional competency and regulation, as well as on the contextual factors that influence the use of picture books. Consequently, there is a gap in context-specific evidence examining teachers’ practices, perceived barriers, and mediating roles in picture book reading activities for social and emotional learning (SEL) within Saudi ECE environments.

The purpose of this study is to address this gap in knowledge by expanding current understanding of how picture books can be employed to support kindergarten children’s emotional awareness, expression, and regulation in Saudi Arabia. This study is aligned with the national education agenda for ECE in Saudi Arabia, which is part of the Saudi Vision 2030 (2021), aimed at developing children’s academic and social skills before they start primary education. Thus, there are four main research objectives of this study, which are as follows:

1. To identify how Saudi educators use picture books as a tool to assist children’s emotional regulation.
2. To identify what emotional themes are most frequently depicted in Saudi children’s picture books.
3. To examine how picture books may be employed to support children’s recognition and understanding of emotions.
4. To identify obstacles that hinder the teachers’ willingness to use picture books for emotional education practices.

To address these objectives, this study aimed to collect Saudi teachers’ views regarding how picture books can be employed to support young children’s emotional awareness, expression, and regulation by exploring the following questions:

1. How do Saudi educators use picture books to support children with emotional regulation?
2. What emotional themes are depicted most frequently in Saudi children’s picture books?
3. In what ways do children benefit from being exposed to picture books regarding emotional awareness?
4. What factors hinder educators’ willingness to use picture books for emotional education?

## Contributions of the study

This study makes several important contributions to the body of research literature on advances in teaching SEL using picture books. Firstly, it provides an insight into the significant role of picture books in supporting emotional development of preschool Saudi children in accordance with the Saudi Vision 2030 and the country’s educational policies. Secondly, this study identifies how picture books are used in Saudi kindergartens, focusing on common pedagogical practices, challenges, and ways to improve emotional reading and learning. Thirdly, this study provides evidence on the importance of picture books in supporting children’s emotional regulation when used in a social context. Fourthly, the study identifies and explains for the first time some significant limitations in the content of picture books used in Saudi kindergartens, namely that books are selected as appropriate for the education system or are modified by publishers to avoid portrayal of negative emotions, such as anxiety, fear, and anger. Thus, the narratives and pictures included in the books neglect important aspects of human emotional expression and, consequently, provide limited support for children in understanding and managing their more confronting emotions. The Saudi cultural factors involved in suppressing negative feelings are explored in this study, leading to a better understanding of how an emphasis on maintaining social harmony, the importance of family honor, and religious beliefs overlooks the need for children to learn about and experience the full range of emotion expression. These cultural observations and findings from the study make a specific contribution and add significant value to knowledge while also presenting serious implications for Saudi teachers and kindergarten administrators as they navigate the complex norms and traditions of Saudi society while providing effective support for children’s emotional development and learning.

## Literature review

### Picture books

Recent academic studies have documented the critical role that picture books play in early childhood education (ECE) and how children’s literature serves as a foundation for young learners (Crawford et al., 2024; Dong et al., 2024; Lepola et al., 2023; Niland, 2023). A picture book is a tool that combines pictures and text to capture children’s interest, as well as introduce fundamental reading skills to children. For example, Crawford et al. (2024) adopted a detective role-play to capture children’s interest as well as facilitating word comprehension, to demonstrate how associations create emotional awareness in a community. This indicates that tools such as pictures, visual symbols, word-image pairings, dialogues, and art activities have a positive effect on language barriers, as well as emotional meaning-making, as long as they are utilized through social interactions. Dong et al. (2024) demonstrated that the use of picture books improved knowledge by 25% for 80 children after utilizing a structured viewing activity, as well as a reflective connection to images. Lepola et al. (2023) also demonstrated that picture books reduced frustration by 50% for different groups after utilizing images as a tool for learning. These studies differentiate between static images as a tool for learning and images that is utilized through social interactions.

Visual emotion symbols, icons, images, and interactive media transform passivity to active emotion mapping, which is critical for early development. In a historical and cultural context, this development occurs through others mediation, witnessing co-constructed emotions socially regulated, and internalized culture tools, which are fundamental components of Vygotsky (1978, 1962) learning theory.

Vivid images are effective in capturing a child’s attention as well as improving language development and comprehension, which provide a collective experience for emotional and social development (Crawford et al., 2024). Picture books allow children to associate words with emotions and relate reading content to real-life situations, which is possible through social interactions and that enable learning environments. Numerous researchers (e.g., Fletcher and Reese, 2005; Grosse et al., 2022; Grøver et al., 2020; Milburn et al., 2014; Schoppmann et al., 2023) identified co-reading picture books as a highly effective and traditional learning strategy for children, which provides enjoyment and learning advantages through facilitating dialogic learning and shared meaning-making.

In collaborative reading sessions, especially those involving dialogic approaches, where teachers use discussion before, during, and after reading, children’s reading and linguistic skills are improved (Dong et al., 2024; Lepola et al., 2023; Milburn et al., 2014; National Institute for Literacy, 2008; Swanson et al., 2011; Walker and Lombrozo, 2017). Evidence supports that children learn from picture books through the content of the books and through mediation by teachers involving questioning and shared interpretation which are main aspects of socio-cultural theory (Vygotsky, 1978).

Splinter et al. (2024) indicated that the use of book covers assist children’s imaginations and lead to engaging conversations. Furthermore, Chen et al. (2023) discovered that teachers use the visual appeal of book covers to initially engage children into conversation. Thus, visuals primarily function as prompts for interaction rather than as stand-alone instructional tools. Additionally, by connecting the stories to the children’s everyday experiences, teachers can encourage children to share and express their thoughts and feelings (Cárdenas et al., 2020; Dickinson, 2001; Halliday, 2004; Strouse et al., 2018). This demonstrates that teachers’ roles include supporting children’s learning while fostering meaning through conversation. Illustrations with stories help children understand and make them more relevant to skill development (Oberman, 2023; Petrová et al., 2025), which is best done by developing discussions and dialogues (Dickinson, 2001; Dickinson et al., 2002; Stewart and Koopmans, 2025; Wells et al., 2022).

Active engagement in read-aloud activities helps to develop vocabulary and reinforce language skills (Senawati et al., 2021; Tjäru, 2023). Research indicates that reading discussions are more effective in facilitating learning transfer from picture books to real life (Strouse et al., 2018; Walker and Lombrozo, 2017; Wang and Shao, 2024). Children retain information better when it is related to their familiarity with images depicted in books (Breitfeld et al., 2021; Callaghan, 2000; Harris et al., 2015; Marjanovic-Umek et al., 2015; Schoppmann et al., 2023; Tare et al., 2010). Familiarity helps to create an anchor for new information, mutual explanation, and appreciation of visual stimuli (Petrová et al., 2025). Thus, picture books enable children to learn information, actions, and words from books as well as from social interactions, which greatly affects learning outcomes.

### Emotions

According to the literature, children experience a broad range of strong and changing emotions during their daily lives (Harper, 2016; Paley and Hajal, 2022; Saarni, 2022; Smith and Pollak, 2020). These emotional responses occur in social contexts that may include conversations with adults and children. It is crucial for children to learn how to identify, express, and understand their own and other children’s feelings (Joseph et al., 2005; Pakarinen et al., 2020; Skura and Świderska, 2021). Learning foundations for social and emotional development starts with emotion recognition, which enables children to express feelings correctly and constructively (Filice and Weese, 2024; Hemmeter et al., 2021). These competencies develop progressively through guided interactions, adult modeling, reflective prompting, and scaffolding of emotional understanding.

Children aged three to six learn to understand and manage their own feelings with increasing understanding, depending heavily on adult support. As children grow older, interactions with parents, teachers, and peers continue to support children’s reasonable management of feelings (Papadopoulos, 2021; Ulutaş et al., 2021; Zinsser et al., 2018). This process reflects the internalization of shared emotional practices, with regulation occurring through social experience and adult facilitation (Vygotsky, 1978).

Guo et al. (2023) proposed that children’s emotional development is contingent on the extension of parental, familial, and educational accountability. Emotional learning thus evolves from an individualistic characteristic to a socially constructed phenomenon that develops over time through interaction. Researchers have also identified the following distinctive characteristics of adult support: Antonopoulou (2024) emphasizes the creation of an inclusive classroom environment that establishes a link with children’s varied emotional needs, while Hemmeter et al. (2021) emphasize the link between preschools and families, and Scott-Barrett et al. (2023) emphasize flexible teaching that provides support for children’s emotional conditions. Collectively, these studies outline complementary layers of adult mediation classroom climate, home–preschool continuity, and adaptive pedagogy.

The capability to cultivate emotional awareness produces numerous benefits, including children’s developing their sense of themselves and their perception of the feelings of others (Arola et al., 2023; Lane and Smith, 2021). Teaching children to identify and describe their feelings improves their language skills and enhances their ability to manage anxiety and negativity, thus reducing antisocial behavior, according to Hemmeter et al. (2021). Knowledge of emotional intelligence serves as a foundation for self-regulation, empathy, and the maintenance of friendly relationships (Antonopoulou, 2024; Berti and Cigala, 2020; Kappelmayer et al., 2022; Romero-Ayuso et al., 2022). Emotional learning increases prosocial behaviors, social confidence, creativity, individuality, strength of self, feelings of belonging and support for increased engagement in classroom activities (Paley and Hajal, 2022; Wesarg-Menzel et al., 2023).

However, Stewart and Koopmans (2025) found that teachers face difficulties in developing emotional learning in children when the school curriculum and teachers’ training do not incorporate emotional competency. This issue gives insight into the gap between the recognition of emotional learning and its implementation capacity. The issue was particularly noted in Saudi Arabia, as highlighted in a study by Kaifi (2023), in which no formal SEL curriculum was found. The issue was noted as requiring teachers’ training in order to improve their knowledge and competency in this area. This issue points towards a restriction rather than a teacher motivation limit for effective SEL use in Saudi preschool settings (Hemmeter et al., 2021; Scott-Barrett et al., 2023).

Dedicated teacher education for social–emotional learning is highly advocated in Western early education settings, given its evidence-based recognition of teachers as agents for learning and development (Soutter, 2023). This view represents a move towards a deliberate pedagogical competence. This was emphasized by Hemmeter et al. (2021) and Scott-Barrett et al. (2023), who noted that effective emotional learning requires both content and teacher capacity to model, demonstrate, and connect to emotions in daily interactions. In addition, pre-service teacher education that includes picture books has been noted to promote children’s emotional development (Daly and Blakeney-Williams, 2015). This highlights the importance of preparing teachers as mediators who use tools, including picture books to scaffold emotional meaning-making and facilitate self-regulation.

### Social–emotional learning in Saudi Arabia

The Kingdom of Saudi Arabia’s Early Learning and Development Standards (ELDS) intend to promote children’s emotional and social development, especially for children aged 3 to 6 years, through developing their skills in emotion regulation and relationship building (Ministry of Education of the Kingdom of Saudi Arabia, 2018). The Ministry’s policy supports children’s emotional, physical, social, and mental growth from birth to age six. However, a study done on the views of preschool educators in Saudi Arabia regarding ELDS noted that SEL does not offer enough guidance information regarding developing emotional and social skills (Alghamdi, 2023; Kaifi, 2023; Ministry of Education of the Kingdom of Saudi Arabia, 2018).

### Saudi Vision 2030 and supporting children’s emotions

The Kingdom of Saudi Arabia’s Saudi Vision 2030 (2021) introduced a new program known as the Human Capability Development Program, which aims to develop a new generation that is founded on emotional intelligence, as well as awareness of different cultures and traditions. Children’s emotional well-being is considered a key factor in developing qualities such as empathy, self-awareness, and emotional control (Armesto Arias et al., 2025). These methods reflect the moral, spiritual, ethical and religious values of Islamic teachings—including those from the Prophet Muhammad, peace be upon him, and the Quran—which are central to Saudi culture. According to the Quran (Surah Aali ‘Imran: 134), Allah Subhanahu wa ta’ala (SWT) loves those who are compassionate and selfless, encouraging believers to embody these prosocial behaviors. These cultural values and religious teachings resonate with modern SEL principles and are essential for Saudi children to learn in early years education.

Alghamdi et al. (2022) also point out that the Vision 2030 links the emotional development of young children with the following main goals for the country: strengthening human capital for the benefits of diversification of the economy, developing social unity by connected culture, developing a globally competitive population while continuing the Islamic identity. The Saudi Vision 2030 (2021), therefore, supports the following: developing access to quality programs, developing teacher training, developing the national curriculum, developing the integration of emotional learning with cultural learning, and developing quality assurance. Nevertheless, the national framework underscores that social and emotional learning and related educational resources should be modified or adapted to align with Saudi traditions and Islamic principles, rather than being implemented in their original Western form without modification.

### Supporting the emotional development of children

Supporting the child’s emotional health is the foundation on which the child’s psychosocial development and learning are based (Qashmer, 2023). Between the ages of 3 to 6 years, the child begins to feel emotions such as joy, sorrow, fear, and anger. Emotional communication can help the child feel a sense of “belongingness,” which is essential in helping the child feel safe and comfortable in the surroundings (Ciesielska et al., 2025). Feeling a sense of belonging to the community gives the child confidence as he discovers the world socially, but a lack of emotional support can cause problems in peer associations and in the child’s ability to cope with life’s challenges in the future (Qashmer, 2023).

Saudi Vision 2030 (2021) has recognized the importance of supporting the child’s emotional health to help the child become a resilient and empathetic member of the family and the community (Dennis et al., 2024; Tamblyn et al., 2024). Picture books can be a good source of support in the development of the child’s emotional health as they provide the child with easy-to-read stories that help the child learn to cope with emotions, especially when the child is encouraged to interact during reading sessions (Aldossary et al., 2021). Therefore, kindergartens provide a safe space for the child to express themselves as the teacher guides them in activities in the classroom to help the child cope emotionally, highlighting the importance of pedagogical mediation in the development of the child’s emotional intelligence.

### Picture books and emotions

Various researchers in childhood development acknowledge the valuable role of picture books in teaching linguistic, social, emotional, and communication skills during teacher-student interactions (Breitfeld et al., 2021; Pulimeno et al., 2020). High-quality picture books can help teachers to teach literacy skills and model best practices in preschool settings by promoting discussions, asking questions, and engaging in collaborative meaning-making (Adam and Barratt-Pugh, 2020; Jalongo, 2004; Oberman, 2023; Zabeli and Gjelaj, 2020).

Picture books help children better understand both their social surroundings and aspects of human nature. By following relatable characters as they encounter common challenges, young readers learn to embrace their emotions, recognize their abilities and limitations, and develop a healthy self-image (Ciesielska et al., 2025; Niland, 2023). By recognizing themselves, they can improve their self- regulation and relationship skills (Breitfeld et al., 2021; M. Chen and Huang, 2025; Wu et al., 2025).

Niland (2023) also argues that picture books can empower teachers to build relationships with students and show them how people cope with real-life problems. Children do not have the experience to cope with the problems they face in the classroom and elsewhere. Therefore, picture books provide them with alternative insights to the problems they face through the stories they read (Green and Sun, 2024; Nikolajeva, 2013). Instead of relying solely on abstract instruction, relatable narrative challenges help children realize others share similar experiences and emotions. When children witness characters overcoming difficulties, classroom discussions guide them toward positive coping strategies (Niland, 2023; Strouse and Ganea, 2021).

The use of visual aids such as facial expressions, body language, spatial arrangements, and composition in picture books aids children in the ability to identify and understand others’ emotions (Kogan, 2024; Strode and Munda, 2023). Teachers who understand the value of these tools and the ability of picture books to help them in the mediation of emotions in the classroom will intentionally incorporate them into lessons (Grosse et al., 2022; Lirola, 2022).

Several studies have supported the notion that children who are supported in their learning are able to manage their emotions well (Catala et al., 2023; Hoel and Jernes, 2023; López-Vallejo et al., 2024; McGrath, 2022). McGrath (2022) noted that the read-aloud picture book sessions in early childhood education provided children with the ability to explore the images and express their thoughts in response to open-ended questions, which helped in the development of the children’s meaning-making abilities with the educators. Children were also able to understand and identify the meanings of words such as “happy,” “sad,” “grumpy,” “afraid,” and “surprised.” These read-aloud sessions helped the children to have an open discussion on their emotions, which gradually helped them in the development of their ability to understand and express their emotions through interaction and discussion, with the story being the focal point of discussion on emotions (Kim and Yim, 2024; Kogan, 2024; McGrath, 2022; Tamblyn et al., 2024; Wu et al., 2025; Xu, 2024). McGrath (2022) noted that the story sessions enhanced children’s ability to express their emotions and helped them to empathize with their peers who were experiencing difficult behaviors.

Picture books can also help the child learn values related to the child’s cultural and religious heritage, as well as instill a sense of community pride in the child (Alghamdi, 2023). By using picture books that feature stories related to Arab or Saudi culture on a regular basis, the home, kindergarten, and library can help the child learn to appreciate their emotional heritage, strengthening their sense of identity and well-being (Alshbili, 2024; Chen et al., 2025).

### Research gap

Although international research in Western contexts highlights the importance of picture books in children’s emotional development, a thorough review of the literature reveals very few studies have explored picture book use in ECE settings outside Western contexts, such as Saudi kindergartens. Several Saudi authors have examined aspects of picture book use, including Al Najim (2024) who studied reading for pleasure, Alsaleh (2019) who considered translation and cultural adaptation of children’s literature, and Asiri (2025) who investigated use of picture books to instill cultural identity in children. Other topics have been researched, including teachers’ perspectives, gender, and death, as depicted in picture books. However, none appear to have examined picture book use for SEL in the Saudi context. Thus, there is limited evidence on how and to what extent Saudi teachers use picture books to support emotional learning in classrooms. To address this knowledge gap, this study investigates how picture books are used in Saudi kindergartens, focusing on common practices, challenges, and ways to improve emotional reading and learning.

## Methods

A self-report questionnaire-based survey was used to collect data, which is useful for the identification of patterns and the statistical verification of the practices of Saudi kindergarten teachers. This is because an understanding of the ways in which different strategies address the challenge is achieved, as themes and strategies tend to overlap (Creswell, 2009).

### Survey instrument

The researcher created the questionnaire based on an exhaustive review of the relevant literature on emotional learning, picture book pedagogy, and sociocultural theory, as they apply to kindergarten education (e.g., Kim and Yim, 2024; Kogan, 2024; McGrath, 2022; Niland, 2023; Strouse and Ganea, 2021). The instrument was theoretically constructed on the basis of four predetermined dimensions; namely, the strategies of the teachers, the emotional themes portrayed in the picture books, the features of the development, and the difficulties faced by teachers. The initial 40 items were created for the questionnaire, addressing the four dimensions. Each item was measured on a five-point Likert scale was provided with options ranging from “strongly agree” to “strongly disagree.”

To test the content validity, the preliminary draft of the questionnaire was evaluated by two experts in ECE and four experts in child psychology. Feedback from the experts on the clarity and appropriateness of the wording of the items, the clarity of the instruction, and the appropriateness of the items in relation to the dimensions was incorporated into the questionnaire. Items that had 90% of the experts’ approval were included in the questionnaire, while five items without 90% experts’ approval were excluded, and five items were deleted, and some items were reworded to improve clarity and relevance, leading to the development of the questionnaire with 35 items.

The survey questionnaire was translated into Arabic, which was the teachers’ preferred language, and the draft was evaluated by a bilingual faculty member at the university to assess whether the semantics were clear and consistent before finalizing the questions. A pilot test was done with several kindergarten teachers to assess several aspects of the program and to ensure the survey is understood and filled out correctly.

The questionnaire’s reliability was evaluated with Cronbach’s alpha and the Spearman–Brown split-half method, showing good internal consistency (alpha 0.724–0.926; overall 0.855) and split-half reliability (0.767–0.862; total 0.861). Since dimensions were theory-based and predefined, validation focused on content validity and internal consistency, not factor analysis.

One of the limitations in this study was the potential methodological bias associated with self-report questionnaires, since participants’ responses might not accurately reflect. For example, social desirability bias might affect the participants to give responses they believe as socially acceptable instead of reflecting their actual practices (de Van Mortel, 2008). Moreover, self-report questionnaires might capture participants’ inaccurate beliefs or recollections instead of observed behavior, which could distort the results (Podsakoff et al., 2003). Therefore, this study sought to minimise bias in questionnaire responses by using clear and concise questions, avoiding leading questions that might suggest a particular answer, and providing participants with sufficient time to think about their responses.

### Construct validity

The construct validity of the questionnaire was verified by calculating the correlation coefficient between the scores of each dimension of the questionnaire and the total questionnaire scores, as shown in Table 1.

**Table 1:** Correlation coefficients between the scores of each dimension and the total questionnaire scores.

Dimensions	Correlation coefficient	Significance level
First dimension	0.569	0.01
Second dimension	0.697	0.01
Third dimension	0.487	0.01
Fourth dimension	0.701	0.01

Table 1 shows a significant correlation (0.487–0.701) between each dimension’s scores and the overall questionnaire, supporting its validity and consistency.

### Questionnaire and dimensions reliability results

Table 2 presents the reliability of the questionnaire and its dimensions, assessed by Cronbach’s Alpha and the Split-Half (Spearman–Brown) Method.

**Table 2:** Reliability coefficients for the questionnaire and its dimensions.

Dimensions	Number of statements	Reliability coefficient	
		Cronbach’s alpha coefficient	Split-half reliability
First dimension	9	0.865	0.862
Second dimension	10	0.826	0.812
Third dimension	10	0.926	0.806
Fourth dimension	6	0.724	0.767
The questionnaire as a whole	35	0.855	0.861

Table 2 shows Cronbach’s Alpha reliability coefficients for the dimensions ranging from 0.724 to 0.926, and 0.855 for the full questionnaire. Split-half coefficients ranged from 0.767 to 0.862, with 0.861 for the whole questionnaire. These values indicate acceptable reliability (Robson, 2002).

### Research ethics

This study received approval from Najran university research ethics committee before the questionnaire was distributed to kindergarten teachers. The teachers consented to participate in the study, and the confidentiality of the data provided was assured, along with their right to withdraw at any time. The names of survey participants were not requested nor were their identities revealed. Participants were assured that their responses were confidential and would only be used for research purposes, as stated in the survey instructions. There was no direct or personal relationship between the researcher and any of the participants. Furthermore, the kindergarten administrative and supervisory bodies of the teachers were not informed whether they had participated in the study and were not advised of teachers’ responses, as indicated in the survey instructions. The instructions also stated that there were no right or wrong answers. Instead, teachers were asked to honestly express their opinions, which they were advised would contribute to the success of the study.

### Research participants

The study participants comprised 113 female kindergarten teachers from Najran city in southwestern Saudi Arabia. All participants for this study were female because the Saudi national education policy currently does not permit males to work as early childhood teachers. The recruitment of teachers from the network of established kindergartens and professional groups took place through digital invitation requests circulated on WhatsApp. Coordination was established with the Kindergarten Education Department in Najran city to identify teachers who met certain criteria. Teachers were included and sent questionnaires based on their specialization in ECE and at least five years of teaching experience. Teachers who were not specialists in the field of childhood education or with less than five years of experience were excluded.

The responses were analyzed using both descriptive and inferential statistics. These included frequencies, means, standard deviations, and chi-square tests to characterize differences and relationships within the four dimensions (Qashmer, 2023). The analyses of these dimensions provided an opportunity to compare the practices of the teachers in relation to four major aspects, namely the teacher’s strategies, emotional themes, developmental features, and challenges. The nature of the kindergarten and access to professional development in emotional education were the contextual factors. The identification of these aspects helped to explain the variations in the application and interpretation of picture books in the various learning situations (Qashmer, 2023).

## Questionnaire survey results

There were four research questions included in the questionnaire with the teachers’ responses to each question being analysed and results reported separately using the same method. To address the research questions, frequencies, mean, standard deviation, relative weight, degree of verification, and ranking were calculated; a Chi-square test (χ^2^) was applied to each statement across four axes and overall (Alford and Teater, 2025). Results are presented in tables with interpretation.

### First research question

The first question asks, “What are the methods of using picture books to help children perceive and manage emotions, from the perspective of the study sample members?”

The results of the first question are shown in Table 3. The statistical significance of the Chi-square (χ^2^) test results at the (0.001) level for all statements of the first axis confirms that the responses were consistent and that there was a clear tendency toward the content of the statements, which enhances the reliability of the obtained results.

**Table 3:** Responses from teachers on how they used picture books to help children coprehend and manage emotions.

No.	Statement	Responses (*n* = 113)					Arithmetic mean	Std. deviation	Relative weight	Degree of Agreement	Rank	χ^2^ value
		Strongly agree	Agreed	Neutral	Disagree	Strongly disagree						
1	I ask guided questions to help children link picture book events to their personal emotions.	33	40	19	12	9	3.67	1.23	7340%.	Agree	3	31.91***
2	I focus on characters’ facial expressions and illustrations to explain emotions to children during storytelling.	13	21	58	15	6	3.18	0.98	6360%.	Neutral	6	74.39***
3	I use tone of voice and emotional acting to convey the emotions included in picture books.	41	32	18	12	10	3.73	1.30	7460%.	Agree	2	31.82***
4	I ask children questions about the emotions and feelings included in picture books.	35	28	30	17	3	3.66	1.15	7320%.	Agree	4	28.90***
5	I present a role-play scene similar to that shown in picture books and allow children to choose the appropriate emotional expression.	19	24	41	15	14	3.17	1.22	6340%.	Neutral	7	21.47***
6	I give children the opportunity to suggest how they would feel if they faced the same events as in the story.	37	33	25	15	3	3.76	1.13	7520%.	Agree	1	33.77***
7	I link story situations to similar real-life situations experienced by children.	15	26	47	17	8	3.20	1.08	6400%.	Neutral	5	40.23***
8	I repeatedly use picture books that address emotions on a regular basis.	18	15	49	18	13	3.06	1.18	6120%.	Neutral	8	39.35***
9	I integrate emotional concepts derived from the story with other daily activities (cooperative play, songs).	21	15	40	22	15	3.04	1.27	6080%.	Neutral	9	18.64***
Overall evaluation of the first axis							3.39	1.21	6780%.	Neutral		

*** Statistically significant at the 0.001 level.

The results of the first axis shown in Table 3 indicate that the level of use of picture books by the participating teachers to help children comprehend and manage emotions was only moderate. The overall arithmetic mean reached 3.39 with a relative weight of 67.80%, corresponding to a neutral degree of agreement. This reflects that the teachers practice some methods of employing picture books to develop children’s emotional awareness; however, these practices were observed to be limited and undeveloped. The results also revealed variation in the degree of use of the different methods. Statements related to giving children opportunities to express their emotions, using tone of voice and emotional acting during reading session, as well as guided questions to help children link picture book events to their personal emotions, obtained relatively higher levels of agreement. This indicates the teachers’ awareness of the importance of direct emotional interaction in conveying emotions to children. In contrast, some methods received a neutral level of agreement, particularly those related to the regular use of picture books, integrating emotion concepts with other daily activities, and linking story situations to similar real-life situations. This suggests that these practices are not yet developed among the participants. The results of this axis reflect a lack of teachers’ awareness of the mechanisms for employing picture books in a more integrated and continuous manner, which would support children’s ability to better exhibit self-regulation.

The second question asks, “What are the topics related to recognizing and managing emotions included in children’s picture books, from the perspective of the study sample members?”

The results of the second question shown in Table 4. The statistical significance of the Chi-square (χ^2^) test results for most statements of the second axis reflects a clear tendency among the teacher toward the content of the statements, which enhances the consistency and credibility of the results.

**Table 4:** Responses on topics related to recognizing and managing emotions included in children’s picture books.

No.	Statement	Responses (*n* = 113)					Arithmetic mean	Std. deviation	Relative weight	Degree of agreement	Rank	χ^2^ value
		Strongly agree	Agree	Neutral	Disagree	Strongly disagree						
10	The stories present a variety of situations that help children understand the differences between emotions.	31	47	27	5	3	3.87	0.96	7740%.	Agree	3	61.03***
11	Some stories focus on recognizing positive emotions (such as gratitude, love, and pride).	33	42	25	7	6	3.79	1.10	7580%.	Agree	5	44.66***
12	The stories include expressive pictures and illustrations that depict facial expressions and body language associated with emotions.	45	39	14	8	7	3.95	1.17	7900%.	Agree	1	57.58***
13	Picture books offer strategies for managing emotions like fear or anxiety (such as breathing exercises and asking for help).	15	17	40	16	25	2.83	1.30	5660%.	Neutral	4	19.52***
14	Picture books present situations that demonstrate how to control anger or cope with rejection and frustration.	19	24	31	22	17	3.05	1.30	6100%.	Neutral	10	5.186
15	Some picture books highlight the importance of verbally expressing feelings.	16	22	45	21	9	3.13	1.12	6260%.	Neutral	7	32.44***
16	Picture books typically include practical solutions for managing emotions.	31	20	35	20	7	3.42	1.24	6840%.	Agree	6	21.29***
17	Picture books present situations that help children understand the emotions of others.	47	34	16	10	6	3.94	1.18	7880%.	Agree	2	53.24***
18	Some picture books demonstrate how to interact with the emotions of others.	20	25	29	25	14	3.11	1.28	6220%.	Neutral	8	5.894
19	Picture books help children understand how their own behavior affects the emotions of those around them.	16	22	43	18	14	3.07	1.19	6140%.	Neutral	9	24.57***
Overall assessment of the second axis							3.42	1.25	6840%.	Agree		

*** Statistically significant at the 0.001 level.

The results of the second axis shown in Table 4 indicate that the topics related to recognizing and managing emotions included in children’s picture books were rated at a relatively high level from the perspective of the teachers. The overall arithmetic mean of the axis reached 3.42 with a relative weight of 68.40%, corresponding to a degree of agreement of “Agree.” This reflects the teachers’ recognition of the importance of picture books in addressing emotion-related aspects for children, particularly when identifying and understanding emotions as part of classroom read-aloud practices. The results also showed that the highest levels of agreement were associated with topics focusing on presenting emotions through expressive pictures and illustrations, helping children understand the emotions of others, and recognizing differences among various emotions. This indicates that teachers regard children’s picture books as appropriate in placing strong emphasis on visual and contextual aspects in presenting emotional concepts. In contrast, some topics received a neutral level of agreement, especially those related to providing clear strategies for overcoming certain negative emotions or managing anger and dealing with frustration. This suggests teachers believe that such topics are not addressed to the same extent in children’s picture books. The results of the second axis indicate teachers believe children’s picture books include, to an appropriate degree, topics related to recognizing and managing emotions, with some variation in the level of emphasis on certain aspects, such as negative emotions.

The third question asks, “What is the role of picture books in supporting children’s ability to understand and manage emotions, from the perspective of the study sample members?”

The results of the third axis shown in Table 5 indicate that the role of picture books in supporting children’s ability to understand and manage emotions was rated highly by teachers. The overall arithmetic mean of the axis reached 3.69 with a relative weight of 73.80%, corresponding to a degree of agreement of “Agree.”

**Table 5:** Teachers’ perspectives on the role of picture books in supporting children’s ability to copmrehend and manage emotions.

No.	Statement	Responses (*n* = 113)					Arithmetic mean	Std. deviation	Relative weight	Degree of Agreement	Rank	χ^2^ value
		Strongly agree	Agree	Neutral	Disagree	Strongly disagree						
20	Picture books help children easily identify their different emotions.	71	23	11	5	3	4.36	1.01	87.20%	Strongly agree	1	140.32***
21	Picture books increase children’s ability to name emotions.	39	16	39	16	3	3.64	1.17	7280%.	Agreed	6	44.66***
22	The expressive illustrations in picture books develop a child’s awareness of the nuances of emotions.	33	22	32	22	4	3.51	1.20	7020%.	Agreed	8	24.04***
23	Picture books help children show concern for and empathy towards the feelings of others.	20	32	23	32	6	3.25	1.20	6500%.	Neutral	10	20.32***
24	Children apply calming strategies they learn from picture books when they are upset.	43	14	40	14	2	3.73	1.15	7460%.	Agreed	4	57.13***
25	Picture books help children manage their anger or anxiety appropriately.	36	15	39	15	8	3.50	1.26	7000%.	Agreed	9	34.39***
26	Conflicts among children decrease after reading picture books about emotional regulation.	37	16	38	16	6	3.55	1.23	7100%.	Agreed	7	35.72***
27	Picture books encourage children to share their feelings with their teacher and peers.	43	31	22	14	3	3.86	1.14	7720%.	Agreed	2	41.82***
28	Picture books about emotions help increase the ability to communicate feelings to others in socially acceptable ways.	41	32	19	15	6	3.77	1.22	7540%.	Agreed	3	34.21***
29	The activities accompanying picture books (drawing/drama) help children express their feelings safely.	42	15	39	15	2	3.71	1.15	7420%.	Agreed	5	52.44***
Overall assessment of the third axis							3.69	1.20	7380%.	Agree		

*** Statistically significant at the 0.001 level.

This indicates the high level of teachers’ awareness of the role of picture books in supporting the development of children’s emotions. Some statements received a high level of agreement; these statements are related to helping children easily identify their feelings, encouraging them to share their feelings with teachers and children, and increasing their ability to express their feelings in a socially acceptable way. This shows teachers believe in the effectiveness of picture books in promoting emotional awareness and emotional communication. However, statements related to children showing interest in and empathizing with the feelings of others have received a neutral level of agreement. This suggests that teachers viewed this empathizing aspect as less influenced by picture books compared to other dimensions of emotional support.

The statistical significance of the Chi-square (χ^2^) test at the (0.001) level for all statements of the third axis reflects a clear and consistent trend in the teachers’ responses confirming their perspectives on the positive role of picture books in supporting children’s ability to comprehend and manage emotions, which enhances the reliability of the results.

The fourth question asks, “What are the difficulties in using picture books to support children’s ability to understand and guide emotions, from the perspective of the study sample members?”

The findings presented in Table 6 demonstrate the statistical significance of the chi-square test results at the 0.001 level for all statements within the fourth axis. This outcome confirms a distinct and consistent trend among research participants regarding the statement content, thereby strengthening the reliability of the results obtained.

**Table 6:** Teachers’ views on difficulties in using picture books to support children’s ability to understand and manage emotions.

No.	Statement	Responses (*n* = 113)					Arithmetic mean	Std. deviation	Relative weight	Degree of agreement	Rank	χ^2^ value
		Strongly agree	Agreed	Neutral	Disagree	Strongly disagree						
30	There is no comprehensive list of children’s emotions that need support at this stage using picture books.	19	25	32	23	14	3.11	1.26	6220%.	Neutral	6	8.02***
31	There is no guide for teachers on how to use picture books about children’s emotions to help children understand their feelings.	29	38	26	11	9	3.59	1.20	7180%.	Agree	4	26.96***
32	Training courses on using picture books related to children’s emotions are unavailable.	18	29	43	16	7	3.31	1.09	6620%.	Neutral	5	33.86***
33	Teachers find it difficult to obtain picture books that help in understanding and managing children’s emotions.	37	19	36	19	2	3.62	1.16	7240%.	Agree	3	37.04***
34	Picture books related to children’s emotions do not cover all the emotions that need to be developed at this stage.	63	32	11	5	2	4.32	0.95	8640%.	Strongly agree	1	114.57***
35	Picture books related to children’s emotions that align with the social and cultural values of Saudi society are not available.	41	37	21	9	5	3.88	1.12	7760%.	Agree	2	46.16***
Overall assessment of the fourth axis							3.64	1.20	7280%.	Agree		

*** Statistically significant at the 0.001 level.

The results of the fourth axis shown in Table 6 indicate that teachers were aware of the difficulties in using picture books to support children’s ability to understand and regulate emotions. The overall arithmetic mean for this axis was 3.64, its relative weight was 72.80%, and the level of agreement was “Agree.” This reflects the teachers’ perceptions of challenges that limit the effectiveness of using picture books in developing children’s emotional aspects. The results also showed that the highest level of agreement was related to the statement that picture books do not cover all the emotions that children need to develop at this stage. This statement ranked first with a high arithmetic mean and a strong level of agreement, indicating that inadequacy in the range of emotional diversity in the picture books represents the most prominent difficulty from the teachers’ perspectives. Teachers also ranked highly the difficulty related to the lack of picture books aligning with the social and cultural values of the Saudi community. Some difficulty statements, especially those about unclear definitions of children’s emotions and the lack of relevant training courses for picture books, drew neutral agreement. This suggests participants differed on how serious these challenges are.

The findings of this axis indicate genuine challenges for teachers in using picture books to help children understand and handle emotions. The next section discusses the findings, priorities, and strategies for integrating these books into the Saudi ECE teaching practices and classroom environment.

## Discussion

This study determined that the Saudi ECE teacher participants considered picture books to be an effective tool for fostering emotional comprehension among students.

### Axis 1: Using picture books to help children understand and manage emotions

Axis 1 emphasized the activities that teachers undertook to impart learning in the areas of comprehension and emotion management, utilizing picture books to assist with the classroom learning activity. The highest-ranking strategies identified by teachers in this study included asking children to imagine how they would feel in the situation portrayed in the story, and using voice modulation and acting during story time to emphasize visual and audible signals that the children could easily follow, asking children questions about the emotions and feelings included in picture books while asking guided questions to help children link picture book events to their personal emotions. These strategies align with the learning techniques found to be effective in prior studies (e.g., Kim and Yim, 2024; Kogan, 2024; McGrath, 2022; Wu et al., 2025), particularly, when teachers employ intense discussion and engagement with children during reading sessions. In these sessions, asking children questions can expand their meaning-making and increase understanding of their emotions. Therefore, the social interaction and connections to discussion are supported by utilizing picture books. Interactive sessions enabled the children to learn feelings from each other and from the teachers, emphasizing that sociocultural context is important in a child’s emotional understanding. These techniques provide interactive opportunities for children to connect a character’s experiences to their own personal experiences, emotions, and feelings, thereby fostering self-awareness and empathy (Nikolajeva, 2013; Niland, 2023). The connection between stories and the emotions of children is similar to the observations of Strouse and Ganea (2021), who indicated that instigating debates during reading improves the capability of children to connect the narratives to real-life experiences. Tamblyn et al. (2024) corroborated these findings, suggesting that teacher-initiated dramatic discussions and interactions are prime moments for emotional growth.

Furthermore, Saudi teachers reported that they did not practice role-playing or reading the text in an emotionally themed way often enough or in a sustained way to help children rehearse and transfer the skills. Other studies (Ciesielska et al., 2025; Kim and Yim, 2024; McGrath, 2022) have shown that implementing lessons using picture books under sustained practice across different contexts cultivates empathy and self-regulation. However, in this study, time constraints and competing curricular demands in the context of Saudi kindergartens may limit or prevent these emotionally supportive reading lesson activities. Professional development may enable teachers to incorporate emotions more frequently into play, music, and cooperative work, thereby reinforcing emotional concepts beyond the story circle and throughout the day. However, sufficient time, opportunity, and motivation is needed to ensure that teachers follow through on any curriculum approaches that utilize picture books in lessons in a sustained and consistent manner (Grosse et al., 2022; Schoppmann et al., 2023). Other studies have also supported these connections, while highlighting the need for innovative approaches to teaching with picture books. For instance, it has been found that questioning strategy is one of the best methods (Xu, 2024), this aligns with the result of this study, which found that guided questions can significantly enhanced emotional regulation scores in children. The author indicated that this improved neural processing of emotional through multimodal engagement. According to sociocultural theory (Vygotsky, 1978), applying scaffolding strategies to support children within their ZPD aids their emotional regulation. In this study, teachers played a mediator and facilitator role during reading sessions to encourage interactions among children that helped scaffold their emotional regulation. Affirming that interactions among the children and teachers is important. Furthermore, it has been found that voice modulation and role-playing by teachers improve empathy development in children (Guldner et al., 2025). In the current study, teachers reported they often use voice modulation and emotion while using read-aloud picture books. Moreover, in the Saudi Arabic context (the language used in preschool teaching), expressive intonation and vocal pitch variation is highly inflected in comparison with English and other languages (Shamsan and Attayib, 2015). Vocal cadence and tonal inflection in speech are important for effective communication in all languages; however, in Arabic grammatical theory, it is essential. Therefore, it is vital for the understanding and language development of Saudi children that teachers practice animated and emotional ways of narrating stories from picture books. Expressive reading can be used to enhance the language growth and story comprehension of Saudi children with the use of picture books (Ciesielska et al., 2025). The teachers themselves play the central role in this process, since they can make the reading interesting by altering the tone of their voice and delivery to portray the various emotions.

The findings of the current investigation emphasize the need to provide Saudi kindergarten teachers with effective professional development, not only to increase the use of picture books, but to advance the pedagogical quality of read-aloud interactions. Teachers’ use of expressive methods, such as vocal modulation, tonality, and accentuation, has a crucial effect on the mediation of children’s comprehension of the story’s meaning. These vocal attributes of the teacher’s reading voice, which comprise paralinguistic features, facilitate children’s ability to identify the characters’ emotions, as well as the story’s changes, and to comprehend the affective content of the story, which might not be directly depicted by the story’s content. Therefore, teachers need to be empowered to use these techniques, which can shift the quality of read-aloud activities from the usual exposure to books to more intentional learning moments that promote children’s emotional engagement and literacy development.

### Axis 2: Topics related to recognizing and managing the emotions included in children’s picture books

Axis 2 examined the emotions that picture books evoke in children during lessons. The teachers strongly agreed that stories using expressive images to portray feelings can help children to recognize the feelings of others. This provides evidence that quality illustrations assist children with understanding feelings through subtle facial and body cues (Crawford et al., 2024; Elliott, 2024). However, from a sociocultural perspective, teachers’ guided discussions and interactions play an important role to mediate emotional meaning during reading aloud sessions.

Additionally, when the visual images used for instruction closely replicate real-life scenarios, children learn more effectively (Breitfeld et al., 2021; Schoppmann et al., 2023). Visual clarity can help children connect complex concepts with their everyday experiences (Niland, 2023). This inclination toward depicting various scenarios in picture books to differentiate emotions is also consistent with Tisnawijaya and Kurniati (2024), who suggested that relatable characters and situations assist children with understanding more complex emotions, such as gratitude or pride. The findings highlight the importance of selecting books that accurately represent the real world through rich, engaging illustrations that can help sustain children’s attention during the read aloud sessions.

In addition to the significance of quality illustrations, the results of the study also showed that the teachers rated the efficacy of teaching strategies such as teaching calming techniques and dealing with others’ emotions as neutral. Therefore, the picture books used in the Saudi Arabian kindergarten settings focus more on emotional recognition than emotional regulation and pay less attention to negative emotions. This is also in line with the research done by Parker and Cronin (2025) regarding children’s books, which showed that picture books usually focus on positive emotions and pay less attention to negative and complex emotions such as fear, rejection, and frustration. In the context of the Saudi Arabian publishing and educational settings, this can also be attributed to the tendency in the cultural settings to prioritize emotional harmony and moral values and socially desirable behavior in the early years. However, various studies (e.g., Kanner, 2024; López-Vallejo et al., 2024; Parker and Cronin, 2025) argued that if children do not get to experience these negative emotions in picture books, they miss the opportunity to learn to cope with unbearable distress.

The picture books used in the Saudi Arabian kindergarten settings can also be understood to be culturally regulated and not simply pedagogically regulated practices. For example, the research done by Alghamdi (2023) showed that the classroom instruction used by the Saudi Arabian preschool teachers is heavily influenced by religion-based and gender-based factors.

Al Najim’s (2024) study of Saudi teachers’ engagement with picture books further revealed that books are often used primarily for curricular and instructional purposes rather than open-ended “reading for pleasure,” which narrows deliberate selection to texts perceived as culturally safe, institutionally approved, and directly teachable. Importantly, the process of selection, translating, and publishing children’s books in Saudi Arabia reinforces these preferences because all literature is subject to licensing and compliance review by the Ministry of Culture and Information. When a foreign book, pictorial, or writing content is imported for use in the Saudi domestic or educational market, translation and adaptation are guided by socio-cultural norms (e.g., religious and ideological constraints) that shape appropriate standards of content (Alsaleh, 2019). Moreover, recent Saudi research has explained how book covers, illustrations, and publisher framing in Saudi Arabia are deliberately used to project and consolidate national-cultural identity in children’s literature (Asiri, 2025). Together, this Saudi-specific evidence supports the view that criteria for selection, translation, and adaptation of literary material for children’s books are centered on cultural–religious alignment, norm-sensitive representation, and institutional and publishing acceptability, alongside developmental appropriateness. These factors help explain the tendency of Saudi children’s books to incorporate limited learning content about negative emotions and behavior, while also accounting for the cultural refining process the books are subject to before being deemed suitable in the Saudi educational context.

In the present study, teachers’ neutral evaluations of items related to coping with fear, anger, and frustration indicate that the picture books currently used in Saudi kindergarten classrooms provide limited support for addressing these emotions. Likewise, Kanner (2024) pointed out that picture books in US tend to give positive emotions more attention over negative or complex emotions. Clearly, there is a need to expand children’s picture books to cover a wide range of emotions themes as there is lake of diversity in picture books emotion’s themes. In another context, Wang et al. (2025) was able to demonstrate a reduction of anxiety distress of preschoolers in China using picture books with a range of positive and negative modelling and culturally applicable content. This highlights a missing opportunity in Saudi kindergartens for children to develop the ability to cope with these negative emotions, such as fear, anger, and frustration in a social context and through teacher mediation.

In contrast, the neutral scores observed in the present study for calming strategies suggest that Saudi classrooms may lack access to locally grounded texts that explicitly model coping with distress. This result aligns with Alharbi et al. (2025) who reported limited improvement in anxiety behaviors among Saudi children following picture-book interventions due at least in part to the lack of cultural fit of the content but also the absence of modelling of the range of anxiety behaviors.

Accordingly, the results of the present study indicate that while picture books support emotional recognition, the development of emotional self-regulation requires culturally responsive content, comprehensive modelling of emotional behaviors, and sustained teacher mediation. This result supports the development of more diverse content in Saudi early learning picture books featuring themes aligned with the ECE aims of Saudi Vision 2030 for the development of citizens who are emotionally resilient, socially skilled, and able to cope with stress (Alharbi et al., 2025).

### Axis 3: The role of picture books in supporting children’s ability to understand and manage emotions

Teachers agreed that the use of picture books assisted children in identifying emotions and promoting sharing. Based on this study’s results, this agreement was strongest for children’s ability to identify emotions and to share their feelings with teachers and peers, indicating that the use of picture books is perceived as particularly effective for emotional recognition and verbal expression. This shows that picture books can stimulate children’s conversation by offering them opportunities to interact and share their emotions with children and teachers, which is consistent with sociocultural theory principles. This confirms the consensus among researchers in the scholarly literature that picture books provide a foundation for children’s emotional development through self-understanding and compassion (Dennis et al., 2024). The role of picture books in implementing calming strategies and reducing conflict was also supported by teachers’ responses, suggesting that the use of picture books can contribute to regulation-related outcomes when used in classroom practice (Tamblyn et al., 2024). However, most of the respondents were neutral in terms of children’s empathy toward the other person in a conflict situation. This might suggest evidence of inconsistencies in implementation, as identified by Pakarinen et al. (2020), who emphasized the importance of adult guidance in fostering higher-order emotional competencies, which aligns with the sociocultural view.

Thus, the use of picture books has been shown to help children understand and manage their feelings, assisting them with learning about emotions, and sharing feelings with peers and teachers, respectively when picture books are used in a social context. From a sociocultural perspective, the present results suggest that these outcomes are most evident in areas that involve immediate interaction, such as naming emotions and communicating feelings rather than in more complex emotional processes. The results are similar to some evidence reported in international studies (e.g., Dennis et al., 2024; Kim and Yim, 2024; Kogan, 2024; McGrath, 2022; Tamblyn et al., 2024; Wu et al., 2025; Xu, 2024) showing that reading aloud develops emotional vocabulary and fosters empathy. However, this study found that empathy-related outcomes were comparatively weaker, demonstrating that exposure to stories is not sufficient for enhancing deeper emotional understanding. According to related studies, illustrations that might express emotion and foster discussions can initiate children’s awareness of the details of emotions and ways to communicate them appropriately (Elliott, 2024; Kogan, 2024). Therefore, sustained social interaction using picture books with teacher mediation in kindergarten classroom sessions is effective in developing empathic concern, which is a key aspect of sociocultural theory.

Teachers in this study also indicated that picture books can encourage children to utilize calming methods and mitigate conflicts. This is consistent with earlier research suggesting that reading aloud and engaging in interactive activities may enhance emotional regulation and foster cooperation (Dennis et al., 2024; Wu et al., 2025). Therefore, these activities enhance emotional learning beyond passive listening by creating shared reading, scaffolding, and guided engagement. Thus, picture books can be used as a teaching tool for SEL in everyday life, including learning about empathy and conflict resolution, when embedded within teacher-facilitated interaction.

Interestingly, according to the teachers in this study, picture books have a greater impact on regulating children’s emotions and resolving conflicts, through conversations and interactions with other children and teachers during reading sessions, than they do on advancing their concern for the feelings of others. This aligns with other studies, such as those by Schoppmann et al. (2023) and Grosse et al. (2022), who argued that, although picture books are effective in improving emotional vocabulary, the development of empathy is not always dependent on them, but rather on the quality of the teacher–child interaction. Teaching empathy effectively requires more than simply being exposed to stories, according to the sociocultural approach, since children learn through discussions, reflective dialog, and shared experiences with others (Kogan, 2024; Qashmer, 2023; Wu et al., 2025). It is teacher facilitation, not only book selection, that also determines the development of children’s deep perspective-taking skills. Therefore, programs that educate teachers to prompt empathy-related dialog can enhance the impact of picture books on prosocial behavior in Saudi kindergartens.

The results regarding empathy were generally moderate, but the analysis performed under Axis 3 revealed a strong relationship between illustrated stories and SEL guidelines, leading to improvements in children’s understanding of the thoughts of other individuals. However, the lower empathy results suggest a need to enhance the picture book themes and contextual content in Saudi kindergarten classrooms. This aligns with the current study recommendations to enhance the visual and thematic choices of the local stories to reflect Saudi cultural values such as family relationships, social responsibility, and pride in heritage thereby supporting Vision 2030 goals related to emotional resilience.

### Axis 4: The challenges of using picture books to support children’s ability to understand and regulate emotions

Axis 4 highlighted many issues that prevent teachers from using picture books optimally. Teachers agreed that current picture books do not cover the full range of emotions children need to develop. Teachers highlighted difficulties in accessing picture books that reflect Saudi culture and values well enough, while the lack of specific curriculum and training guidance for SEL practice using picture books is also challenging.

This shortcoming related to the lack of appropriate cultural content and guidelines for using picture books is not unique to the Saudi kindergarten education system. A survey of international literature on the subject of culture in children’s books by UNESCO (2024), in partnership with the *International Journal of Early Childhood*, discovered that local cultural information and relevance was missing or inadequate in 70% of children’s educational books. The present study results align with this international pattern but also indicate that Saudi teachers perceive this limitation as a challenge to their support of children’s emotional growth. As indicated by previous researchers, such as Alghamdi (2023) and Alshbili (2024), there is a lack of culturally relevant and comprehensive children’s literature available in the Saudi education system, while authors also suggest teacher preparation and professional development in social–emotional pedagogy is also lacking. The strong agreement observed among the teachers in the current study suggests that these issues persist and have not yet been adequately addressed through policy or resource provision.

Insufficient training and professional development opportunities were also identified in this study as barriers to progress. Teachers noted that they were willing and motivated to teach SEL using picture books but had few workshops and little guidance on how to incorporate emotional aspects into their daily practice. Inadequate training means that effective practices, such as consistent and sustained reading of emotional themes or cross-curriculum teacher collaboration, will not occur as frequently as required for the desired outcomes. This helps explain why teachers in this study identified difficulties in accessing suitable materials and guidance, despite expressing strong motivation to support emotional learning.

The findings of this study demonstrate that picture books are more than literacy resources; they are also important tools for enhancing emotional intelligence, communication skills, and cultural competency. However, the present study shows that the effectiveness of teachers in the use of picture books is constrained by inadequacies in cultural relevance, emotional coverage, and teacher guidance in picture books available to Saudi teachers. The study provides timely and nation-specific information relevant to picture books and the social–emotional learning literature in the Saudi context suggesting that more effective practices can be achieved by aligning classroom practices with the nationwide educational agenda (Tamblyn et al., 2024). Overall, the findings emphasize that emotional learning through picture books is not hindered by teacher willingness, but by structural and cultural limitations in resources and training. In line with the sociocultural perspective, teachers play an important role as a mediator to scaffold children’s growth by offering the support that children need to understand their emotions. Therefore, teacher training is important to prepare them with the skills they need to facilitate interactions among children, support children’s feelings, and use tools like picture books to assist children’s emotional development.

## Conclusion and implications

This study has investigated the use of picture books as practical tools in promoting the emotional development of Saudi kindergarten children. The Saudi kindergarten teachers who participated in this research provided insights and clarification concerning their teaching practices and views on utilizing picture books in early learning education. According to the results of the research, guided questions, reading the picture books in emotionally expressive ways, and animated illustrations helped children identify, label, understand, and express their own feelings. This confirms research findings from other studies regarding the effectiveness of shared reading and dialogic interaction, demonstrating the importance of the sociocultural setting in a child’s emotional development (Dennis et al., 2024; Kogan, 2024; Tamblyn et al., 2024). Children’s picture books do not simply tell a story; they also convey a message. They can also impart important skills to children, such as empathy, by enabling them to recognize and identify the emotions of others using facial cues (Strouse and Ganea, 2021). Improvement in other valuable early skills, including self-control and cooperative play with others, is also promoted by picture books (Breitfeld et al., 2021; Chen and Huang, 2025; Niland, 2023). The available research data confirm that picture books not only offer literacy support but are also a valuable resource that can be applied to promote socio-emotional learning in young children (Breitfeld et al., 2021; Chen and Huang, 2025; Niland, 2023). The results further point to significant deficiencies that need to be addressed to enable picture books to reach their full potential. The participants reported that they lacked culturally appropriate titles for the classroom, and that they struggled to access professional development or source clear guidance from the curriculum on how to use picture books to facilitate children’s emotional learning. These findings are consistent with several recent studies (e.g., Alghamdi, 2023; Alshbili, 2024) which have suggested that barriers exist in Saudi teacher education and in the selection of culturally appropriate teaching materials for kindergarten classrooms.

The findings of this study have significant policy implications. The MOE’s recent documents—the ELDS (2018) and Early Education Policy (2021) under Saudi Vision 2030—emphasise social and emotional development. Vision 2030 aims for citizens to be socially rooted and emotionally stable. Integrating picture books and resources into kindergartens can help achieve these goals. Collaboration among curriculum designers, teachers, and resource creators is therefore essential for developing materials that support SEL in classrooms and advance national objectives.

## Limitations, recommendations, and future research

### Limitations

The study’s findings are subject to several limitations. In this sample, only 113 female kindergarten teachers were chosen from one city in Saudi Arabia, which restricts the study to this particular context and limits its generalizability with other populations, such as male teachers or teachers of other education levels from other cultural backgrounds. Furthermore, some bias may have arisen from the willingness of the teachers to participate since the teachers self-selected their participation in the study instead of being chosen randomly. Self-report surveys were used in the data collection, which may not accurately reflect actual classroom practices; rather, they may indicate the participants’ personal perceptions. Since the research was conducted within a short time frame, the cross-sectional form of research provided a snapshot of the current situation; however, it is unlikely to illuminate the long-term effects on the emotional growth of children. A more diverse and larger sample size, as well as direct, qualitative observations in the classroom, may have enabled a more accurate evaluation of the impact of picture books on students’ emotional development over time.

### Recommendations

This study recommends a number of key steps to enhance the effectiveness of teachers in using picture books to support children’s emotional understanding and expression in the Saudi kindergarten environment:

1. Curriculum designers and kindergarten administrators should collaborate with kindergarten teachers to make available a broad range of culturally relevant picture books reflecting complex emotions, such as anxiety and emotional distress, thus enabling children to engage with and learn coping mechanisms for difficult experiences they may encounter.
2. Kindergarten teachers should be offered targeted training in expressive reading, emotional discussion, and vocabulary skills development in read-aloud picture book lessons, as well as interactive strategies such as dialogic reading, voice modulation, and role-playing.
3. The Ministry of Education should develop national social–emotional learning guidelines linked to use of picture books for consistent ECE classroom practice, together with mechanisms for collaboration among educators, picture book publishers, and policymakers.

These measures would help address current inadequacies in SEL instruction in Saudi kindergartens and align with Vision 2030 social and developmental goals, while supporting cultural identity through education and the use of picture books that reflect Saudi and Islamic values (Alghamdi et al., 2022; Saudi Vision 2030, 2021).

### Future research

Future research could build on this study by using a mixed methods approach, which would expand a quantitative survey with semi-structured and open-ended interviews, using qualitative observations and child assessments to verify teacher perceptions. Teachers’ attitudes and practices could be examined in conjunction with training, professional development interventions and observation of classroom practices. Further, children’s progress in emotional development using picture books could be tracked through longitudinal studies, based on their continuing progress in primary and secondary education. To validate the effect of varied reading practices on empathy and emotional competence, an experimental or integrated plan could be employed to compare the impact of dialogic reading-aloud with that of conventional reading-aloud (Wu et al., 2025). Finally, a survey on parents and children participating in shared picture book reading at home would be an ideal way to determine the effect of family participation on realizing the educational and emotional learning aims of Saudi Vision 2030.

### Scope for SEL in Saudi Arabia’s research landscape

Saudi Arabia provides a robust environment for research in education and social emotional learning. This is largely due to the strong policy support offered by the Saudi Vision 2030 initiative, which prioritizes research and innovation across the nation. Vision 2030 aims to promote socio-economic diversification and ensure sustainability, while also working to transform Saudi Arabia into a leader in global research. These supportive policies create a dynamic landscape that is advantageous to researchers and ambitious research projects. Given this context, the present study encourages students and scholars to leverage the opportunities available in Saudi Arabia by taking advantage of this conducive environment to pursue projects that make meaningful contributions to the nation’s 21st century goals and objectives.

## Data Availability

The original contributions presented in the study are included in the article/supplementary material, further inquiries can be directed to the corresponding author.

## References

[ref1] Adam H. Barratt-Pugh C. (2020). The challenge of monoculturalism: what books are educators sharing with children and what messages do they send? Aust. Educ. Res. 47, 815–836. doi: 10.1007/s13384-019-00375-7

[ref1001] Alford S. Teater B. (2025). Quantitative research. In Handbook of research methods in social work, Edward Elgar Publishing. 156–171.

[ref2] Al Najim A. S. (2024). Exploring Student Teachers’ reading Habits and the Use of Picturebooks in the Kindergarten Setting in Saudi Arabia (Glasgow, United Kingdom: Doctoral thesis, University of Glasgow).

[ref3] Aldossary N. Curwood J. S. Niland A. (2021). Fostering multilingual children’slanguage development through iPad apps. Read. Teach. 75, 329–338. doi: 10.1002/trtr.2051

[ref4] Alghamdi A. A. (2023). Culture in early childhood education: insights into Saudi preschool teaching. J. Educ. Learn. 17, 431–440. doi: 10.11591/edulearn.v17i3.20804

[ref5] Alghamdi A. K. H. Alsaadi R. K. Alwadey A. A. Najdi E. A. (2022). Saudi Arabia’s Vision 2030’s compatibility with women and children’s contributions to national development. Interchange 53, 193–214. doi: 10.1007/s10780-021-09451-3

[ref6] Alharbi T. M. Roslan S. Aap N. A. A. (2025). Social competence among children with mild intellectual disabilities in Saudi Arabia: the role of self-confidence and failure expectation. Int. J. Acad. Res. Prog. Educ. Dev. 14, 1078–1094. doi: 10.6007/IJARPED/v14-i3/26114

[ref7] Alsaleh A. H. M. (2019). The Position and Norms of Translated Children’s Literature in Saudi Arabia: A Multimodal socio-Cultural Approach. (Leeds, United Kingdom: Doctoral thesis, University of Leeds).

[ref8] Alshbili N. (2024). Saudi Kindergarten Teachers’ Perceptions of Teacher–child Interaction Quality before and after a Professional Development Initiative. (Dublin, Ireland: Doctoral Dissertation, Dublin City University).

[ref9] Antonopoulou H. (2024). The value of emotional intelligence: self-awareness, self-regulation, motivation, and empathy as key components. Technium Education Humanities 8, 78–92. doi: 10.47577/teh.v8i.9719

[ref10] Armesto Arias M. Neira-Piñeiro M. D. R. Pasarin-Lavin T. Rodríguez C. (2025). A drama-based intervention to improve emotional intelligence in early childhood education. Eur. J. Psychol. Educ. 40:13. doi: 10.1007/s10212-024-00906-6

[ref11] Arola T. Aulake M. Ott A. Lindholm M. Kouvonen P. Virtanen P. . (2023). The impacts of nature connectedness on children’s well-being: systematic literature review. J. Environ. Psychol. 85:101913. doi: 10.1016/j.jenvp.2022.101913

[ref12] Asiri E. (2025). Constructing Saudi cultural identity through paratext: a case study of the translated children’s book Sidra’s adventure in AlUla. J. King Abdulaziz Univ. Arts Humanit. 33, 548–563. doi: 10.4197/Art.33-1.20

[ref13] Barrett L. F. Adolphs R. Marsella S. Martinez A. M. Pollak S. D. (2019). Emotional expressions reconsidered: challenges to inferring emotion from human facial movements. Psychol. Sci. Public Interest 20, 1–68. doi: 10.1177/1529100619832930, 31313636 PMC6640856

[ref14] Berti S. Cigala A. (2020). Mindfulness for preschoolers: effects on prosocial behavior, self-regulation and perspective taking. Early Educ. Dev. 33, 38–57. doi: 10.1080/10409289.2020.1857990

[ref15] Breitfeld E. Potter C. E. Lew-Williams C. (2021). Children simultaneously learn multiple dimensions of information during shared book reading. J. Cogn. Dev. 22, 744–766. doi: 10.1080/15248372.2021.1939353, 34744519 PMC8570620

[ref17] Callaghan T. C. (2000). Factors affecting children’s graphic symbol use in the third year. Cogn. Dev. 15, 185–214. doi: 10.1016/s0885-2014(00)00026-5

[ref18] Cárdenas K. Moreno-Núñez A. Miranda-Zapata E. (2020). Shared book-reading in early childhood education: teachers’ mediation in children’s communicative development. Front. Psychol. 11:2030. doi: 10.3389/fpsyg.2020.02030, 33013511 PMC7505735

[ref19] Catala A. Gijlers H. Visser I. M. (2023). Guidance in storytelling tables supports emotional development in kindergartners. Multimed. Tools Appl. 82, 12907–12937. doi: 10.1007/s11042-022-14049-7

[ref20] Chen T.-I. Chung H.-J. Lin S.-K. (2023). The effect of applying language picture books in reciprocal teaching on students’ language learning motivations. SAGE Open 13:21582440231218857. doi: 10.1177/21582440231218857

[ref21] Chen M. Huang Y.-C. (2025). Analysis on the role of picture books in children’s cognitive development education. Edelweiss Applied Science Technology 9, 1916–1925. doi: 10.55214/25768484.v9i3.5718

[ref22] Chen H. Lyu D. Zhu L. (2025). The effectiveness of social-themed picture book reading in promoting children’s prosocial behavior. Front. Psychol. 16:1569925. doi: 10.3389/fpsyg.2025.1569925, 40297596 PMC12034717

[ref23] Ciesielska M. Kucirkova N. Thomson J. (2025). How the type and context of children's storybook reading relate to select empathy skills: a meta-analysis. Early Educ. Dev. 36, 1888–1914. doi: 10.1080/10409289.2025.2516989

[ref24] Cordeiro T. Botelho J. Mendonça C. (2021). Relationship between the self-concept of children and their ability to recognize emotions in others. Front. Psychol. 12:672919. doi: 10.3389/fpsyg.2021.672919, 34712163 PMC8545983

[ref25] Crawford P. A. Roberts S. K. Lacina J. (2024). Picture books and young children: potential, power, and practices. Early Child. Educ. J. 52, 1273–1279. doi: 10.1007/s10643-024-01701-0

[ref26] Creswell J. (2009). Research design: Qualitative, Quantitative, and Mixed Methods Approach. 3rd Edn London, England: SAGE Publications, Ltd.

[ref27] Cruz S. Sousa M. Mateus V. (2024). “Emotion regulation and cognitive and social functioning in early development: the interface between neurophysiological and behavioural perspectives,” in Emotional Intelligence—Understanding, Influencing, and Utilizing Emotions, ed. Laurent E. (London, United Kingdom: IntechOpen).

[ref28] Daly N. Blakeney-Williams M. M. (2015). Picturebooks in teacher education: eight teacher educators share their practice. Aust. J. Teach. Educ. 40, 88–101. doi: 10.14221/ajte.2014v40n3.6

[ref29] de Van Mortel T. F. (2008). Faking it: social desirability response bias in self-report research. Aust. J. Adv. Nurs. 25, 40–48.

[ref31] Dennis L. Wade T. Cole T. Morsching D. Haglund C. (2024). The effects of shared book reading on the emotional vocabulary development of preschool children. Vocabulary Learn. Instr. 13, 1–22. doi: 10.29140/vli.v13n2.1186

[ref32] Dickinson D. K. (2001). “Book reading in preschool classrooms: is recommended practice common?” in Beginning Literacy with Language, eds. Dickinson D. K. Tabors P. O. (Baltimore, Maryland, United States: Paul H. Brookes Publishing), 175–203.

[ref33] Dickinson D. K. McCabe A. Anastasopoulos L. (2002). A Framework for Examining book reading in Early Childhood Classrooms, CIEARA Report #1–014. Ann Arbor, Michigan, United States: Center for the Improvement of Early Reading Achievement, University of Michigan School of Education.

[ref34] Dong Y. Chow B. W. Mo J. Miao X. Zheng H. (2024). Effects of dialogic reading elements on children’s language development. J. Res. Read. 47, 181–200. doi: 10.1111/1467-9817.12447

[ref35] Elliott L. M. (2024). Applied Behavior Analysis picture to text word Comprehension Intervention for children with Moderate to Severe Impairments due to Autism spectrum Disorder. (Buffalo, New York, United States: Doctoral Dissertation, State University of New York at Buffalo).

[ref36] Filice L. Weese W. J. (2024). Developing emotional intelligence. Encyclopedia 4, 583–599. doi: 10.3390/encyclopedia4010037

[ref37] Fletcher K. L. Reese E. (2005). Picture book reading with young children: a conceptual framework. Dev. Rev. 25, 64–103. doi: 10.1016/j.dr.2004.08.009

[ref39] Greber H. Lecheler S. Aaldering L. De Haan Y. Kruikemeier S. Goutier N. . (2023). Feeling the news? The differential effects of immersive journalism on emotional response. Digit. Journal. 11, 39–60. doi: 10.1080/21670811.2022.2155205

[ref40] Green C. Sun H. (2024). Picture books increase the frequency and diversity of emotion vocabulary in children’s language environments: modeling potential benefits to emotional literacy, with pedagogical resources. Early Educ. Dev. 36, 568–586. doi: 10.1080/10409289.2024.2423259

[ref41] Grosse G. Simon A. Soemer A. Schönfeld R. Barth S. Linde N. (2022). Teacher–child interaction quality fosters working memory and social-emotional behavior in two-and-three-year-old children. Int. J. Early Child. 54, 421–444. doi: 10.1007/s13158-022-00327-w

[ref42] Grøver V. Rydland V. Gustafsson J. Snow C. E. (2020). Shared book reading in preschool supports bilingual children’s second-language learning: a cluster-randomized trial. Child Dev. 91, 2192–2210. doi: 10.1111/cdev.13348, 31943173

[ref43] Guldner S. Nees F. Flor H. McGettigan C. (2025). Speaker differences in volitional voice modulation reflected in empathy and functional activation patterns. PLoS One 20:e0325207. doi: 10.1371/journal.pone.0325207, 40720533 PMC12303263

[ref44] Guo J. Tang X. Marsh H. W. Parker P. Basarkod G. Sahdra B. . (2023). The roles of social–emotional skills in students’ academic and life success: a multi-informant and multicohort perspective. J. Pers. Soc. Psychol. 124, 1079–1110. doi: 10.1037/pspp0000426, 35666915

[ref45] Halliday M. A. K. (2004). “The place of dialogue in children’s construction of meaning,” in Theoretical Models and Processes of reading, eds. Ruddell R. B. Unrau N. J.. 5th *ed* (Newark, Delaware, United States: International Reading Association), 133–145.

[ref46] Harper L. J. (2016). Preschool through primary grades: using picture books to promote social-emotional literacy. YC Young Children 71, 80–86.

[ref47] Harris C. Straker L. Pollock C. Smith A. (2015). Children, computer exposure and musculoskeletal outcomes: the development of pathway models for school and home computer-related musculoskeletal outcomes. Ergonomics 58, 1611–1623. doi: 10.1080/00140139.2015.1035762, 25942433

[ref48] Hemmeter M. L. Ostrosky M. Fox L. (2021). Unpacking the pyramid model: A Practical guide for Preschool Teachers. Baltimore, Maryland, United States: Paul H. Brookes Publishing Co.

[ref49] Hoel T. Jernes M. (2023). Quality in children’s digital picture books: seven key strands for educational reflections for shared dialogue-based reading in early childhood settings. Early Years 44, 480–494. doi: 10.1080/09575146.2023.2172552

[ref50] Jalongo M. R. (2004). Young children and picture Books. Washington, DC, United States: National Association for the Education of Young Children.

[ref52] Joseph G. Strain P. Ostrosky M. M.. (2005). Fostering emotional literacy in young children: Labeling emotions. Center on the Social and Emotional Foundations for Early Learning. Available online at: https://csefel.vanderbilt.edu/briefs/wwb21.pdf (Accessed April 10, 2026).

[ref53] Kachel G. Hardecker D. J. K. Bohn M. (2021). Young children’s developing ability to integrate gestural and emotional cues. J. Exp. Child Psychol. 201:104984. doi: 10.1016/j.jecp.2020.104984, 33038706

[ref54] Kaifi A. I.. (2023). Saudi Arabian Preschool Teachers’ Perspectives of the need for Socialemotional Learning Curriculum. (River Forest, Illinois, United States: Doctoral Dissertation, Concordia University Chicago).

[ref55] Kanner S. C.. (2024). Read-Alouds for social-Emotional Learning in Preschool: A Mixed-Methods Study [Doctoral Dissertation, St. John’s University].

[ref56] Kappelmayer M. Czar A. Tresca M. D’Adamo P. Lozada M. (2022). A school intervention promotes compassion, empathy and social relationships in children. School Psychol. Int. 44, 515–532. doi: 10.1177/01430343221145668

[ref57] Kim W. J. Yim D. (2024). Exploring the influence of the home literacy environment on early literacy and vocabulary skills in Korean–English bilingual children. Front. Psychol. 15:1336292. doi: 10.3389/fpsyg.2024.1336292, 38524291 PMC10958979

[ref58] Kleye I. Sundler A. J. Darcy L. Karlsson K. Hedén L. (2021). Children’s communication of emotional cues and concerns during a preoperative needle procedure. Patient Educ. Couns. 105, 1518–1523. doi: 10.1016/j.pec.2021.09.035, 34625321

[ref59] Kogan A. M. (2024). Using dialogic reading and direct instruction of emotion words to increase emotion vocabulary knowledge in the preschool classroom. J. Research Innovative Teaching Learning 17, 201–219. doi: 10.1108/JRIT-12-2023-0192

[ref60] Lane R. D. Smith R. (2021). Levels of emotional awareness: theory and measurement of a socio-emotional skill. J. Intelligence 9:42. doi: 10.3390/jintelligence9030042, 34449662 PMC8395748

[ref61] Lanza K. Alcazar M. Chen B. Kohl H. W. (2023). Connection to nature is associated with social-emotional learning of children. Curr. Res. Ecol. Soc. Psychol. 4:100083. doi: 10.1016/j.cresp.2022.100083

[ref63] Lepola J. Kajamies A. Laakkonen E. Collins M. F. (2023). Opportunities to talk matter in shared reading: the mediating roles of children’s engagement and verbal participation in narrative listening comprehension. Early Educ. Dev. 34, 1896–1918. doi: 10.1080/10409289.2023.2188865

[ref64] Lirola M. M. (2022). Compositional and interpersonal analysis of the picture book Stella brings the family: deconstructing affection. Onomázein 55, 1–23. doi: 10.7764/onomazein.55.01

[ref65] Liu J. Green R. J. (2024). Children’s pro-environmental behaviour: a systematic review of the literature. Resour. Conserv. Recycl. 205:107524. doi: 10.1016/j.resconrec.2024.107524

[ref6565] LoBue V. Ogren M. (2022). How the emotional environment shapes the emotional life of the child. Policy Insights Behavi Brain Sci. 9, 137–144. 10.1177/23727322211067264 PMC9435752 36059861

[ref66] López-Vallejo S. Burneo-Garcés C. Pérez-García M. (2024). Development of working memory and inhibitory control in early childhood: cross-sectional analysis by age intervals and gender in Ecuadorian preschoolers. PLoS One 19:e0299394. doi: 10.1371/journal.pone.0299394, 38743790 PMC11093310

[ref67] Malik F. Marwaha R. (2022). Developmental stages of social-emotional development in children. StatPearls. 30521240

[ref68] Marcin A. (2020). Reading to children: why it’s so important and how to start. Healthline.

[ref69] Marjanovic-Umek L. Fekonja-Peklaj U. Sočan G. Tašner V. (2015). A socio-cultural perspective on children’s early language: a family study. Eur. Early Child. Educ. Res. J. 23, 69–85. doi: 10.1080/1350293x.2014.991096

[ref71] McGrath E. (2022). “How does engagement with picture books impact on the development of young children’s emotional literacy?” in Children’s Literature in action, eds. Charlesworth R. Friedland D. Jones H. (London, United Kingdom: Gold Publishing), 124–143.

[ref72] Milburn T. F. Girolametto L. Weitzman E. Greenberg J. (2014). Enhancing preschool educators’ ability to facilitate conversations during shared book reading. J. Early Child. Lit. 14, 105–140. doi: 10.1177/1468798413478261

[ref73] Ministry of Education of the Kingdom of Saudi Arabia (2018). National Early Learning Framework for Children ages 0–6. Washington, DC, United States: National Association for the Education of Young Children.

[ref1177] National Institute for Literacy (2008). Developing early literacy: Report of the National Early Literacy Panel. National Institute for Literacy. Available online at: https://lincs.ed.gov/publications/pdf/NELPReport09.pdf

[ref74] Nikolajeva M. (2013). Picture books and emotional literacy. Read. Teach. 67, 249–254. doi: 10.1002/trtr.1229

[ref75] Niland A. (2023). Picture books, imagination and play: pathways to positive reading identities for young children. Educ. Sci. 13:511. doi: 10.3390/educsci13050511

[ref76] Oberman R. (2023). From invitation to destination: a systematic literature review of the use of picture books in inquiry-based education. Br. Educ. Res. J. 49, 555–574. doi: 10.1002/berj.3856

[ref78] Pakarinen E. Lerkkanen M. K. von Suchodoletz A. (2020). Teacher emotional support in relation to social competence in preschool classrooms. Int. J. Res. Method Educ. 43, 444–460. doi: 10.1080/1743727X.2020.1791815

[ref79] Paley B. Hajal N. J. (2022). Conceptualizing emotion regulation and coregulation as family-level phenomena. Clin. Child. Fam. Psychol. Rev. 25, 19–43. doi: 10.1007/s10567-022-00378-4, 35098427 PMC8801237

[ref80] Papadopoulos D. (2021). Parenting the exceptional social-emotional needs of gifted and talented children: what do we know? Children 8:953. doi: 10.3390/children8110953, 34828666 PMC8624036

[ref81] Parker F. Cronin J. (2025). Fostering social-emotional learning in children's books: an analysis of content and quality. Ga. J. Lit. 47, 24–45. doi: 10.56887/galiteracy.191

[ref83] Petrová Z. J. Vašíková J. Žáková I. (2025). “I’m just looking at the pictures…”: the role of illustrations in books from the perspective of preschool children. J. Res. Child. Educ., 1–16. doi: 10.1080/02568543.2025.2461563

[ref84] Podsakoff P. M. MacKenzie S. B. Lee J.-Y. Podsakoff N. P. (2003). Common method biases in behavioral research: a critical review of the literature and recommended remedies. J. Appl. Psychol. 88, 879–903. doi: 10.1037/0021-9010.88.5.879, 14516251

[ref85] Pulimeno M. Piscitelli P. Colazzo S. (2020). Children’s literature to promote students’ global development and wellbeing. Health Promotion Perspectives 10, 13–23. doi: 10.15171/hpp.2020.05, 32104653 PMC7036210

[ref86] Qashmer A. F. (2023). Emotion regulation among 4–6-year-old children and its association with their peer relationships in Jordan. Front. Psychol. 14:1180223. doi: 10.3389/fpsyg.2023.1180223, 37744599 PMC10516536

[ref87] Rangel-Rodríguez G. A. Badia M. Blanch S. (2021). Encouraging emotional conversations in children with complex communication needs: an observational case study. Front. Psychol. 12:674755. doi: 10.3389/fpsyg.2021.674755, 34295286 PMC8290146

[ref89] Robson C. (2002). Real World Research. 2nd Edn Oxford, England: Blackwell.

[ref90] Romero-Ayuso D. Espinosa-García B. Gómez-Marín E. Gómez-Jara N. Cuevas-Delgado C. Álvarez-Benítez I. . (2022). A pilot study of improving self-regulation and social interaction with peers: an “exciting school.”. Children (Basel). 9:829. doi: 10.3390/children9060829, 35740766 PMC9222160

[ref91] Saarni C. (2022). Emotional development in childhood. Encyclopedia on Early Childhood Development. Available online at: https://www.child-encyclopedia.com/emotions/according-experts/emotional-development-childhood

[ref92] Sanchis-Sanchis A. Grau M. D. Moliner A.-R. Morales-Murillo c. p. (2020). Effects of age and gender in emotion regulation of children and adolescents. Front. Psychol. 11:946. doi: 10.3389/fpsyg.2020.00946, 32528367 PMC7265134

[ref118] Saudi Vision 2030. (2021). Saudi Vision 2030: Kingdom of Saudi Arabia. Government of Saudi Arabia. Available online at: https://www.vision2030.gov.sa

[ref93] Schoppmann J. Severin F. Schneider S. Seehagen S. (2023). The effect of picture book reading on young children’s use of an emotion regulation strategy. PLoS One 18:e0289403. doi: 10.1371/journal.pone.0289403, 37531357 PMC10395841

[ref94] Scott-Barrett J. Johnston S.-K. Denton-Calabrese T. McGrane J. A. Hopfenbeck T. N. (2023). Nurturing curiosity and creativity in primary school classrooms. Teach. Teach. Educ. 135:104356. doi: 10.1016/j.tate.2023.104356

[ref95] Senawati J. Suwastini N. K. A. Jayantini I. G. A. S. R. Adnyani N. L. P. S. Artini N. N. (2021). The benefits of reading aloud for children: a review in EFL context. IJEE 1, 73–100. doi: 10.15408/ijee.v1i1.19880

[ref96] Shamsan M. A. H. A. Attayib A. M. (2015). Inflectional morphology in Arabic and English: a contrastive study. Int. J. Engl. Linguist. 5, 139–150. doi: 10.5539/ijel.v5n2p139

[ref97] Shoshani A. (2024). The roots of compassion in early childhood: relationships between theory of mind and attachment representations with empathic concern and prosocial behavior. J. Exp. Child Psychol. 242:105880. doi: 10.1016/j.jecp.2024.105880, 38368743

[ref98] Silkenbeumer J. Lüken L. M. Holodynski M. Kärtner J. (2024). Emotion socialization in early childhood education and care–how preschool teachers support children's emotion regulation. Social Emotional Learning 4:100057. doi: 10.1016/j.sel.2024.100057

[ref99] Skura M. Świderska J. (2021). The role of teachers’ emotional intelligence and social competences with special educational needs students. Eur. J. Spec. Needs Educ. 37, 1–16. doi: 10.1080/08856257.2021.1885177

[ref100] Smith K. E. Pollak S. D. (2020). Early life stress and development: potential mechanisms for adverse outcomes. J. Neurodev. Disord. 12:34. doi: 10.1186/s11689-020-09337-y, 33327939 PMC7745388

[ref101] Song S. (2024). Evaluating the extent to which the colors used in the illustrations in children’s picture books have a positive effect on the intellectual development of young children. Int. J. Comput. Sci. Inf. Technol. 4, 157–159. doi: 10.62051/ijcsit.v4n3.16

[ref102] Soutter M. (2023). Transformative social-emotional learning for teachers: critical and holistic well-being as a marker of success. J. Teach. Learn. 17, 7–30. doi: 10.22329/jtl.v17i1.7001

[ref103] Splinter S. E. Depaepe F. Verschaffel L. Torbeyns J. (2024). A teacher’s choice: preschool teachers’ selection and use of picture books for mathematics instruction. Early Child Res. Q. 66, 135–146. doi: 10.1016/j.ecresq.2023.10.002

[ref104] Stewart C. Koopmans H. (2025). Critical picture book literacy. J. Vis. Literacy 44, 71–93. doi: 10.1080/1051144x.2025.2458444

[ref105] Strode A. Munda L. (2023). “Illustrations of expressions of emotions in children’s books,” in Human, Technologies and Quality of Education 2023, (Riga, Latvia: University of Latvia Press), 594–603.

[ref106] Strouse G. A. Ganea P. A. (2021). The effect of object similarity and alignment of examples on children’s learning and transfer from picture books. J. Exp. Child Psychol. 203:105041. doi: 10.1016/j.jecp.2020.105041, 33279828

[ref107] Strouse G. A. Nyhout A. Ganea P. A. (2018). The role of book features in young children’s transfer of information from picture books to real-world contexts. Front. Psychol. 9, 1–14. doi: 10.3389/fpsyg.2018.00050, 29467690 PMC5807901

[ref109] Swanson E. Vaughn S. Wanzek J. Petscher Y. Heckert J. Cavanaugh C. . (2011). A synthesis of read-aloud interventions on early reading outcomes among preschool through third graders at risk for reading difficulties. J. Learn. Disabil. 44, 258–275. doi: 10.1177/0022219410378444, 21521868 PMC3319370

[ref110] Tamblyn A. Sun Y. North A. Godsman N. Boothby C. Skouteris H. . (2024). Educators’ perspectives on the role of the early childhood environment in supporting children’s social and emotional development. Australas. J. Early Child. 49, 140–154. doi: 10.1177/18369391231221560

[ref111] Tan L. Volling B. L. Gonzalez R. LaBounty J. Rosenberg L. (2022). Growth in emotion understanding across early childhood: a cohort-sequential model of firstborn children across the transition to siblinghood. Child Dev. 93, e299–e314. doi: 10.1111/cdev.13729, 34970992 PMC9851428

[ref112] Tare M. Chiong C. Ganea P. DeLoache J. (2010). Less is more: how manipulative features affect children’s learning from picture books. J. Appl. Dev. Psychol. 31, 395–400. doi: 10.1016/j.appdev.2010.06.005, 20948970 PMC2952631

[ref113] Tisnawijaya C. Kurniati G. (2024). Anna Kang’s picture books: inculcating young minds with social-emotional literacy. PRO 18, 306–317.

[ref114] Tjäru S. (2023). Bolstering and bridging—pre-primary teachers’ purposes and views of reading aloud. Nord. J. Literacy Res. 9, 1–19. doi: 10.23865/njlr.v9.5107

[ref115] Twiner A. Lucassen M. Tatlow-Golden M. (2022). Supporting children’s understanding around emotions through creative, dance-based movement: a pilot study. Learn. Cult. Soc. Interact. 37:100659. doi: 10.1016/j.lcsi.2022.100659

[ref116] Ulutaş İ. Engin K. Polat E. B. (2021). “Strategies to develop emotional intelligence in early childhood,” in The Science of Emotional Intelligence, ed. Taukeni S. G. (London, United Kingdom: IntechOpen).

[ref117] UNESCO (2024). Cultural gaps in children’s literature: a global survey of emotional learning resources. Int. J. Early Child. 56, 145–162.

[ref1199] Vygotsky L. S. (1962). Thought and Language. MIT Press.

[ref119] Vygotsky L. S. (1978). Mind in Society: The Development of Higher Psychological Processes. Cambridge, Massachusetts, United States: Harvard University Press.

[ref120] Walker C. M. Lombrozo T. (2017). Explaining the moral of the story. Cognition 167, 266–281. doi: 10.1016/j.cognition.2016.11.007, 28010879

[ref121] Wang L. Cui R. Wan N. Hu W. (2025). Reshaping the ability–strategy link in emotion regulation: the role of a structured picture-book intervention for preschoolers. Behav. Sci. 15:1137. doi: 10.3390/bs15081137, 40867494 PMC12383865

[ref122] Wang Z. Shao Y. (2024). Picture book reading improves children’s learning understanding. British J. Development Psychology 43, 12–35. doi: 10.1111/bjdp.12479, 38415288 PMC11823313

[ref123] Wang J. Z. Zhao S. Wu C. Adams R. B. Newman M. G. Shafir T. . (2023). Unlocking the emotional world of visual media: an overview of the science, research, and impact of understanding emotion. Proc. IEEE 111, 1236–1286. doi: 10.1109/JPROC.2023.3273517, 37859667 PMC10586271

[ref124] Weir K. (2023). How to Help Kids Understand and Manage Their Emotions. Washington, DC, United States: American Psychological Association.

[ref125] Wells M. S. Morrison J. D. López-Robertson J. (2022). Building critical reading and critical literacy with picturebook analysis. Read. Teach. 76, 191–200. doi: 10.1002/trtr.2130

[ref126] Wesarg-Menzel C. Ebbes R. Hensums M. Wagemaker E. Zaharieva M. S. Staaks J. P. C. . (2023). Development and socialization of self-regulation from infancy to adolescence: a meta-review differentiating between self-regulatory abilities, goals, and motivation. Dev. Rev. 69:101090. doi: 10.1016/j.dr.2023.101090

[ref127] Whitburn J. Abrahamse W. Linklater W. L. (2023). Do environmental education field trips strengthen children’s connection to nature and promote environmental behaviour or well-being? Current Research Ecological Social Psychology 5:100163. doi: 10.1016/j.cresp.2023.100163

[ref128] Wu Y. Chen S. Wang X. Qiao P. Jiang Y. (2025). Teacher-child talk in shared-book reading with preschoolers: linkages between teacher questioning and child responsiveness. Early Child. Educ. J. 53, 33–45. doi: 10.1007/s10643-024-01830-6

[ref129] Xu J. (2024). Developing emotional intelligence in children through dialogic reading, self-made books, and visible thinking routines. Early Child. Educ. J. 52, 1693–1705. doi: 10.1007/s10643-023-01520-9

[ref130] Zabeli N. Gjelaj M. (2020). Preschool teacher’s awareness, attitudes and challenges towards inclusive early childhood education: a qualitative study. Cogent Educ. 7:1791560. doi: 10.1080/2331186x.2020.1791560

[ref132] Zinsser K. M. Denham S. A. Curby T. W. (2018). Becoming a social and emotional teacher the heart of good guidance. YC Young Children 73, 77–83.

[ref133] Zinsser K. M. Gordon R. A. Jiang X. (2021). Parents’ socialization of preschool-aged children's emotion skills: a meta-analysis using an emotion-focused parenting practices framework. Early Child Res. Q. 55, 377–390. doi: 10.1016/j.ecresq.2021.02.001

